# Robot-assisted laparoscopic low anterior resection for rectal cancer with persistent descending mesocolon: A case report

**DOI:** 10.1016/j.ijscr.2022.106793

**Published:** 2022-01-24

**Authors:** Kenjiro Hirai, Jun Takeshima, Jun Ichikawa, Haruku Fujita, Kosuke Toda, Akira Mitsuyoshi

**Affiliations:** Department of Surgery, Otsu City Hospital, 2-9-9 Motomiya, Otsu City, Shiga 520-0804, Japan

**Keywords:** PDM, persistent descending mesocolon, RLAR, robot-assisted laparoscopic low anterior resection, CT, computed tomography, IMA, inferior mesenteric artery, LCA, left colic artery, S1, sigmoid artery first branch, IMV, inferior mesenteric vein, Persistent descending mesocolon, Robot-assisted surgery, Colorectal cancer

## Abstract

**Introduction:**

Persistent descending mesocolon (PDM) is a fixed abnormality in which the descending to sigmoid colon adheres to the small intestinal mesentery or right pelvic wall through right displacement. Surgery for colorectal cancer with PDM is difficult. Therefore, in addition to the anatomical characteristics of PDM, the extent of adhesion and characteristics of vascular courses need to be assessed in individual patients. The number of patients now undergoing laparoscopic or robot-assisted surgery for colorectal cancer has rapidly increased. We herein report a rectal cancer patient with PDM who safely underwent robot-assisted laparoscopic low anterior resection (RLAR).

**Presentation of case:**

A 71-year-old male was referred to our hospital for a detailed examination following a fecal occult blood-positive reaction. Lower gastrointestinal endoscopy revealed a type 2 lesion of the rectum. Moderately differentiated adenocarcinoma was diagnosed based on the results of a histopathological examination. Preoperative contrast-enhanced thoracoabdominal computed tomography showed abnormalities in the colonic course and characteristic vascular courses, suggesting rectal cancer with PDM. RLAR was performed.

**Discussion:**

In surgery, it is important to initially perform adhesiolysis accurately in order to reconstruct the original shape of the colonic mesentery and confirm/dissect vascular bifurcations due to the risk of marginal arterial injury.

**Conclusion:**

In the present case, a detailed anatomical understanding of the site of intestinal adhesion and vascular courses, as well as surgical procedures, facilitated safe RLAR. We described this case and reviewed the anatomical characteristics of PDM and cautions for surgery.

## Highlights

• The descending colon is not fused to the retroperitoneum in persistent descending mesocolon.
• In colorectal cancer with persistent descending mesocolon, surgery is difficult.
• Adhesiolysis is performed for original colonic mesentery shape recovery.
• Due to the risk of marginal artery injury, vascular bifurcations are dissected.
• Robot-assisted laparoscopic low anterior resection is safe for rectal cancer with persistent descending mesocolon.

## 1 Introduction

Persistent descending mesocolon (PDM) refers to a fixed abnormality in which the descending to sigmoid colon adheres to the small intestinal mesentery or right pelvic wall through right displacement [1], [2]. PDM is not problematic in laparotomy and may be managed. However, strategies for surgical procedures, such as anatomical recognition and adhesiolysis, are required in laparoscopic or robot-assisted surgery on colorectal cancer patients with PDM. The number of patients now undergoing laparoscopic surgery for colorectal cancer has rapidly increased. Previous studies performed laparoscopic colectomy in the presence of PDM [3], [4], [5], [6], whereas few conducted robot-assisted surgery [7], [8]. Robot-assisted laparoscopic low anterior resection (RLAR) was safely performed on the present case of rectal cancer with PDM. We reviewed the anatomical characteristics of PDM and cautions for surgery.

This case report has been reported in line with the Surgical CAse REport (SCARE) guidelines [9].

## 2 Presentation of case

The patient was a 71-year-old male with a height of 167 cm and body weight of 62 kg. He had no remarkable symptoms; however, the fecal occult blood test was positive in a health check-up. Lower gastrointestinal endoscopy revealed a type 2 lesion of the upper rectum. Biopsy findings suggested moderately differentiated adenocarcinoma. Therefore, the patient was referred to our department for surgery.

**Fig. 1:**
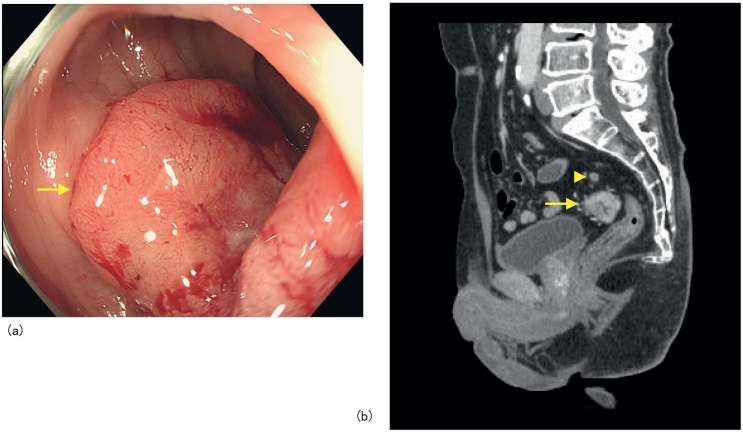
Diagnosis of a primary focus. a) Lower gastrointestinal endoscopy findings: A type 2 rectal tumor was observed (arrow). b) Contrast-enhanced thoracoabdominal CT findings (sagittal section): In the rectum, wall thickening with contrast effects (arrow) and regional lymph node swelling (arrow head) were noted.

The medical and family histories of the patient were not contributory. A blood examination showed that the levels of the tumor markers CEA and CA19-9 were within normal ranges at 3.1 ng/mL and 13 U/mL, respectively. Lower gastrointestinal endoscopy revealed a type 2 lesion measuring approximately 30 mm in the rectum 13 cm from the anal margin. Biopsy findings suggested moderately differentiated adenocarcinoma (Fig. 1a).

**Fig. 2:**
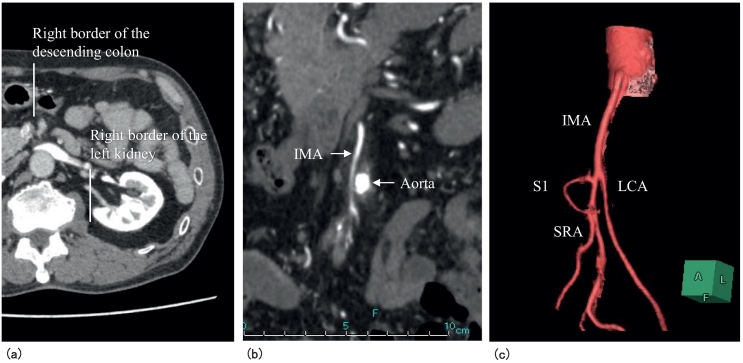
Diagnosis of PDM (contrast-enhanced thoracoabdominal CT). a) Horizontal section: The right margin of the descending colon was located on the right of the medial margin of the left kidney. b) Coronal section: The IMA course involved the right of the abdominal aorta. c) 3D angiography: The LCA, S1, and SRA radially branched at the same position from the IMA.

Wall thickening with contrast effects and regional lymph node swelling were noted in the upper rectum on contrast-enhanced thoracoabdominal computed tomography (CT) (Fig. 1b). Right displacement of the descending/sigmoid colon was also observed. The course of the inferior mesenteric artery (IMA) involved the right of the abdominal aorta, and the left colic artery (LCA), sigmoid artery first branch (S1), and superior rectal artery (SRA) radially branched from the IMA (Fig. 2a, b, and c). Rectal cancer (cT3N1M0, stage IIIB, 8th Union for International Cancer Control classification) with PDM was diagnosed based on these findings and RLAR was performed.

**Fig. 3:**
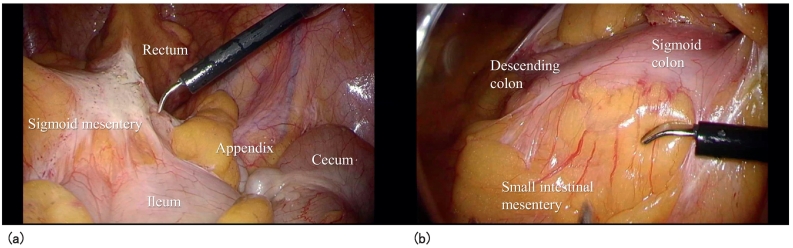
Intraoperative findings. a) The medial sigmoid colonic mesentery adhered to the small intestinal mesentery. b) The sigmoid colon was short and linear until the descending colon was reached. The medial side adhered to the small intestinal mesentery.

**Fig. 4:**
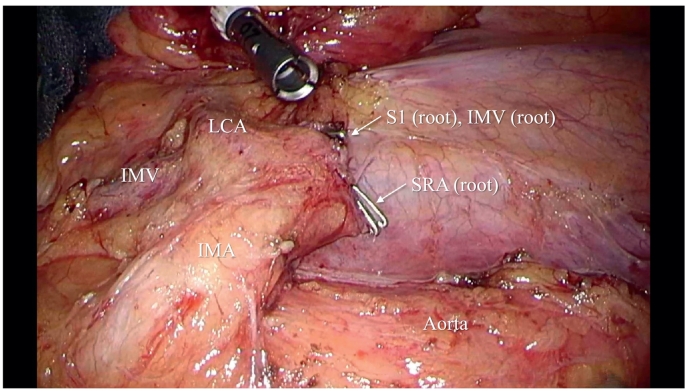
Intraoperative findings. The LCA was preserved, and the SRA, S1, and IMV were dissected.

Right displacement of the descending to sigmoid colon with extensive adhesion to the small intestinal mesentery was noted during surgery (Fig. 3a and b). After careful exfoliation of the site of the sigmoid/descending colonic adhesion to reconstruct the original shape of the colonic mesentery, a medial approach was initiated through the right rectum. Lymph node No. 253 was dissected. The LCA was preserved for vascular treatment, and the S1 and SRA were separated (Fig. 4). The inferior mesenteric vein (IMV) was separated at the same level. After mobilization of the rectum, a specimen was extirpated using a 5-cm area on the anal side of the tumor as the separation line. The intestinal tract was reconstructed using the double stapling technique. A hybrid method with a laparoscope and the da Vinci® surgical Si system (Intuitive Surgical Inc.) was adopted. Adhesiolysis and intestinal tract reconstruction were performed by laparoscopy. The operative time was 276 min and the blood loss volume was small.

The postoperative course was favorable and the patient was discharged 7 days after surgery. Histopathological findings suggested pT3N1M0 stage IIIB.

## 3 Discussion

Positional abnormalities of the colon occur through fixed abnormalities of the mesentery in the embryonic phase; however, positional abnormalities are rarer for the left colon than for the right colon. After 5 months of gestation, the ascending and descending colonic mesentery assimilates to the parietal peritoneum and is then permanently fixed to the retroperitoneum [2], [3]. PDM is a condition in which the descending colonic mesentery does not assimilate to the posterior/lateral parietal peritoneum in this developmental process. A radiodiagnosis of PDM is based on the following: 1) the right margin of the descending colon is medial to the right margin of the left kidney, 2) the IMA course involves the right of the abdominal aorta, and 3) IMA vessels branch at the same position, showing a radial course. The incidence of PDM is reportedly 2.3% [7]. In the present case, CT showed the above findings, and PDM was diagnosed before surgery.

Minimally invasive surgery, such as laparoscopic or robot-assisted surgery, is now increasingly performed; however, surgery for fixed abnormalities of the colon, such as PDM, is difficult. Since intraperitoneal observations are limited during robot-assisted surgery, the anatomical misrecognition of or damage to other organs may occur due to fixed abnormalities or adhesion. In the present case, robot-assisted surgery was performed by rolling in a da Vinci® system after adhesiolysis by laparoscopy.

As a PDM strategy for mobilization of the intestinal tract or lymph node dissection, the following 3 anatomical characteristics need to be identified [3]: (1) the course of the colon and extent of adhesion, (2) radial vascular bifurcations, and (3) shortening of the colonic mesentery.

PDM may be evaluated based on the adhesion of the sigmoid colon to the medial small intestinal mesentery or colonic mesentery when the small intestine is eliminated to the cephalic side. In PDM-free patients, the position of the IMA may be readily estimated by confirming the Treitz ligament or IMV. However, difficulties are associated with the confirmation of these landmarks in PDM patients due to extensive adhesion of the mesentery; therefore, adhesiolysis must initially be conducted. The site of sigmoid colonic adhesion needs to be carefully removed in order to reconstruct the original shape of the colonic mesentery. When the layer to be exfoliated at the site of adhesion is not clear, observations from various angles, particularly the dorsal side, provide a better field of view. In the present case, the sigmoid colon was short and linear. However, a long sigmoid colon, showing an “N”-shaped adhesion between the sigmoid colon and descending colon, has been reported [3]. Although the sufficient removal of adhesions is difficult and time-consuming in these cases, it is important for preventing tension at the site of anastomosis. The completion of medial adhesiolysis facilitates IMA holding/traction and is followed by a standard medial approach. The IMA pedicle is short in many patients with PDM, making it challenging to develop the mesentery to the ventral side. However, the connective tissue plexus on the dorsal side of the rectum may be visually recognized by holding the dissected margin of the sigmoid colonic mesentery.

A standard IMA bifurcation rises from the aorta and branches to the LCA and then to the S1. The SRA reaches the intrapelvic cavity. However, in patients with PDM, the distance between the LCA and S1 bifurcations is short, and radial branching is observed in many cases [4], [5], including the present case. Furthermore, the descending colonic mesentery is often markedly shortened; therefore, marginal artery injury may occur when the course of a marginal artery is adjacent to the IMA or LCA [7]. To ensure safe intestinal anastomosis, the morphology of bifurcations needs to be sufficiently assessed and the blood vessels to be treated are then selected based on the site of intestinal resection. Briefly, when dissecting the IMA at its root, the sigmoid colon needs to be dissected until an area with favorable blood flow is reached. In contrast, when preserving the sigmoid colon, it is safer to preserve the LCA by clarifying the complex morphology of bifurcations [3], [5], [6]. The balance between intestinal/vascular dissection sites and lymph node dissection is influenced by the tumor site or stage. However, in the present case, the LCA was preserved and the SRA was dissected due to the risk of marginal artery injury after IMA dissection. Extracorporeal dissection also needs to be considered when it is difficult to evaluate the intraperitoneal anatomy [7]. Furthermore, an assessment of intestinal blood flow using the indocyanine green fluorescence method is useful to ensure safe anastomosis [5].

Surgery was safely performed on the present case due to a detailed understanding of the anatomical characteristics of PDM and carefully conducting the procedures involved. When left colectomy or rectectomy is performed in the presence of PDM, the site of adhesion needs to be initially and accurately removed in order to reconstruct the original shape of the colonic mesentery. Furthermore, vascular bifurcations need to be confirmed/dissected due to the risk of marginal artery injury.

The operative time of laparoscopic colectomy for colorectal cancer was significantly longer in patients with PDM than in PDM-free patients, while the blood loss volume was slightly larger. PDM has been identified as a risk factor for unsuccessful laparoscopic surgery [4] due to the difficulties associated with extensive adhesiolysis and evaluations of the anatomical positional relationship. Limited information is currently available on the safety of robot-assisted surgery for PDM due to the small number of case reports; however, PDM may be a risk factor for unsuccessful robot-assisted surgery, similar to laparoscopic surgery. We herein safely performed surgery using laparoscopy. The further accumulation of cases is needed to obtain evidence for the safety of robot-assisted surgery for PDM.

## 4 Conclusion

We safely performed RLAR for rectal cancer with PDM. A detailed understanding of anatomical characteristics, such as the site of adhesion and vascular courses, and strategies for surgical procedures facilitated safe robot-assisted surgery.

## Funding

This research did not receive any specific grant from funding agencies in the public, commercial, or not-for-profit sectors.

## Ethics approval and consent to participate

The present study was conducted in accordance with the ethical standards of our institution.

## Consent for publication

Written informed consent was obtained from the patient for publication of this case report and accompanying images. A copy of the written consent is available for review by the Editor-in-Chief of this journal on request.

## Author contributions

KH contributed to the writing of the manuscript. KH, JT, JI, HF, and KT performed surgery and postoperative care. AM contributed to the writing of the manuscript as well as complete supervision. All authors read and approved the final manuscript.

## Registration of research studies

This research was registered with the Research Registry UIN (www.researchregistry.com). Registration ID: 7460.

## Guarantor

Kenjiro Hirai: kenjiro@kuhp.kyoto-u.ac.jp.

## Provenance and peer review

Not commissioned, externally peer-reviewed.

## Declaration of competing interest

None.

## References

[1] Morgenstern L. (1960). Persistent descending mesocolon. Surg. Gynecol. Obstet..

[2] Popky G.L., Lapayowker M.S. (1966). Persistent descending mesocolon. Radiology.

[3] Okada I., Yamaguchi S., Kondo H., Suwa H., Tashiro J., Ishii T. (2013). Laparoscopic colectomy for persistent descending mesocolon: an experience of 13 patients. J. Jpn. Soc. Endosc. Surg..

[4] Wang L., Kondo H., Hirano Y., Ishii T., Hara K., Obara N. (2020). Persistent descending mesocolon as a key risk factor in laparoscopic colorectal cancer surgery. In Vivo.

[5] Hiyoshi Y., Miyamoto Y., Eto K., Nagai Y., Iwatsuki M., Iwagami S. (2019). Laparoscopic surgery for colorectal cancer with persistent descending mesocolon. World J. Surg. Oncol..

[6] Furuichi Y., Kumamoto K., Asano E., Kondo A., Uemura J., Suto H. (2020). Four cases of laparoscopic colectomy for sigmoid colon and rectal cancer with persistent descending mesocolon. Surg. Case Rep..

[7] Hanaoka M., Hino H., Shiomi A., Kagawa H., Manabe S., Yamaoka Y. (2021). Minimally invasive surgery for colorectal cancer with persistent descending mesocolon: radiological findings and short-term outcomes. Surg. Endosc..

[8] Y Kojima K Sakamoto M Kawai Y Okazawa K Kure A Kobari Laparoscopic surgery and robot-assisted surgery for colorectal cancer with persistent descending mesocolon. https://doi.org/10.21203/rs.3.rs-63249/v1.

[9] Agha R.A., Franchi T., Sohrabi C., Mathew G., Kerwan A., SCARE Group (2020). The SCARE 2020 guideline: updating consensus Surgical CAse REport (SCARE) guidelines. Int. J. Surg..

